# Supplementary material for: Research on the impact effect of multimodal transport on domestic and international dual circulation: Evidence from China’s railway and water transport
Source: PLoS One. 2025 Apr 25;20(4):e0319982. doi: 10.1371/journal.pone.0319982 (PMC12026926; doi:10.1371/journal.pone.0319982)
Supplement: Attachment — Submitted filename: Response to Reviewers.docx [file pone.0319982.s003.docx]

Dear Editor：

We are very grateful to your and the reviewers’ critical comments and thoughtful suggestions. Based on these comments and suggestions, we have made careful modification on the original manuscript. All changes made to the text are in red in the revised manuscript so that they may be easily identified. Some of your questions were answered below.

Once again, we acknowledge your comments and constructive suggestions very much, which are valuable in improving the quality of our manuscript.

Here are our responses to the reviewers’ comments one-by-one.

**Ⅰ. Reply to the comments of the first review expert**

1. It is suggested to add consecutive line numbers.

**We added consecutive line numbers.**

2.  In terms of literature review, the distinction between the research content of existing references is not clear enough. It is recommended to expand the number of references and try to highlight the innovative points of this paper in tables or other forms.

**We increased the number of references, made the classification of literature review more complete and clear, and expounded the innovative points of the paper more prominently in the paper. (2 Literature review)**

3.  In section 4.1, the explanation of the parameters and variables in formula (1) does not correspond to the formula.
1) In the subscript, which does i represent, the province or the year?

**In the subscript, i represents the province and t represents the year. We have marked it in the original text.**

2) Please explain α0, α1, and α2 separately.

 **is the intercept term,****、** **all are coefficients, that is, the specific impact of multimodal transport on the domestic and international double cycle.**

3) β1, β2, Zit, and subsequent symbols do not appear in formula (1).

**The three symbols are writing errors. We are sorry for our carelessness and have corrected them in the article.**

4. In section 4.2.1, the text mentions "further open China's coastal areas by developing labor-intensive and export-oriented processing businesses". China's coastal areas are relatively developed, and in recent years, they have been upgrading their industries. Is this statement inappropriate?

**The statement of "further opening up the coastal areas" has been corrected in the article, and the development of international external circulation can continue to expand the market scale and further introduce foreign investment to develop the economy.**

5. Both 4.2.1 and 4.2.2 involve explanatory variables, it is suggested to handle them together.

**The variables in section 4.2.1 are explained variables, that is, dependent variables in the regression model; The variable in section 4.2.2 is the core explanatory variable, that is, the independent variable in the regression model. The previous expression was not clear enough and has now been modified.**

6.  In section 5.3, the random-effects model is mentioned. Does the random-effects model used in the case have a specific expression? In terms of the dual circulation issue of domestic and international, which model is more suitable, the fixed-effects model or the random-effects model?

**The expression of the random effects model has been added to the section 5.3 Robustness test. Before choosing whether to use the fixed effects model or the random effects model for regression, we first conducted the Hausmann test. After the test, the experimental data were more suitable for the fixed effects model for regression, so the fixed effects model was chosen as the main regression model in this paper.**

7.  In section 5.3, why is tail reduction used to remove extreme data, is it because the data structure is similar to a queue or a stack?

**There may be some errors in the experimental data we selected, so we choose the method of tail reduction to remove extreme data. The tail reduction carried out in this paper means to find 2.5% and 97.5% of the extreme value of the data, and then replace the number less than 2.5% with the number at 2.5%, and replace the number greater than 97.5% with the number at 97.5%, and the original data is directly changed into the new data.**

8. In section 5.4, Table 9 mentions the eastern, central, and western regions. It is suggested to provide the criteria and results for the division of these regions.

**According to the documents of the National Development and Reform Commission of China, the research samples were divided into eastern, central and western regions. Among them, eastern and western regions were divided according to the social and economic development status of different regions in China. The eastern region included Beijing, Fujian, Guangdong, Hainan, Hebei, Jiangsu, Liaoning, Shandong, Shanghai, Tianjin and Zhejiang. The central region includes Anhui, Heilongjiang, Henan, Hubei, Hunan, Jiangxi, Jilin and Shanxi, while the western region includes Gansu, Guangxi, Chongqing, Guizhou, Inner Mongolia, Ningxia, Qinghai, Shaanxi, Sichuan, Xinjiang, Yunnan and Tibet. Relevant content has been added in the article.**

9.  In section 6.2, it is suggested to subdivide policy suggestions to correspond to the detailed items in the conclusions of section 6.1, in order to highlight the contributions of the paper.

**The content of policy recommendations has been supplemented according to the opinions of experts, and the conclusion of the paper corresponds to the policy recommendations one by one.**

**Ⅱ. Reply to the comments of the second review expert**

1. It is recommended to add more references, summarize the deficiencies in existing research as well as the innovation points of the current study.

**We increased the number of references, made the classification of literature review more complete and clear, and expounded the innovative points of the paper more prominently in the paper. (2 Literature review)**

2. Why opt for a fixed effects model? What models are commonly employed in existing research? Compared to existing models, what advantages does your proposed model have?

**Before choosing the fixed effects model or the random effects model for regression, we first conducted the Hausmann test. After the test, the experimental data were more suitable for the fixed effects model for regression, so the fixed effects model was chosen as the main regression model in this paper.**

3. The explanation of the model symbols does not correspond to the formula. Please provide a detailed explanation of each component in the model and the reasons for selecting these factors.

**We have revised and interpreted the symbols of the model, in which,  Yit is the explained variable, representing the development level of domestic and international dual-circulation in**$i$**provinces and**$t$**years;****is the intercept term;****、****are coefficients, represent the specific impacts of multimodal transport on domestic and international dual-circulation; Xit is the core explanatory variable, represents the level of multimodal transport in different provinces and years;****represents control variables, including regional economic development level, government support, employment population, and per capita disposable income;**$pro$**represents the provincial effect,**$year$**represents the year effect, and****represents the error term.**

4. Could you elucidate the process of regression analysis, bridging the model with the case study in detail?

**The processing of model and real case data and the specific links have been added to section 4.1，“The dependent variables of domestic large cycle and international external cycle change with the change of multimodal transport and time, and the statistical data are in units of years. Is the intercept term,****is the conditional average when Xit =0, that is, Yit , the development level of domestic and international double circulation when the level of multimodal transport is 0. For the purpose of observation,****represents the impact of multimodal transport on the domestic and international double cycle.”**

5.  It is necessary for the manuscript to enumerate the provinces in the eastern, central, and western regions.

**According to the documents of the National Development and Reform Commission of China, the research samples were divided into eastern, central and western regions. Among them, eastern and western regions were divided according to the social and economic development status of different regions in China. The eastern region included Beijing, Fujian, Guangdong, Hainan, Hebei, Jiangsu, Liaoning, Shandong, Shanghai, Tianjin and Zhejiang. The central region includes Anhui, Heilongjiang, Henan, Hubei, Hunan, Jiangxi, Jilin and Shanxi, while the western region includes Gansu, Guangxi, Chongqing, Guizhou, Inner Mongolia, Ningxia, Qinghai, Shaanxi, Sichuan, Xinjiang, Yunnan and Tibet. Relevant content has been added in the article.**

6. Could you discuss the limitations of this study and suggest potential avenues for future research? Are there any areas that need further investigation?

**Based on your comments and those of other reviewers, we have revised the limitations and future research directions of this paper to section 6.1.“This paper also has some limitations, leaving room for future research. Firstly, multimodal transport started relatively late in China, with few indicators are available to measure its development level, and the data published by national statistical departments are relatively limited. Therefore, more indicators and data can be explored in future research to enrich the evaluation system of multimodal transport. Secondly, multimodal transport may have a deeper impact on the domestic and international dual-circulation system, and the relationship may not necessarily be a simple linear one. Specific effects can be regressed using other models. In future research, we will pay more attention to the more specific impacts of multimodal transport on the domestic and international dual-circulation system, and further clarify the relationship between them.”**

**Ⅲ. Reply to the comments of the third review expert**

1. In this paper, there are too many long sentences and subordinate clauses, and these sentences should be split for better reading. Moreover, some names, provinces and grammatical expressions are not appropriate, such as "General Secretary Xi Jinping", "Shaanxi Province" (Shaanxi Province, China), and "the specific impacts" (the specific impact). It is recommended that the authors make a professional touch-up of the paper.

**For the problems mentioned by experts, we have revised them and added country restrictions to the nouns with regional characteristics.**

2. 3.Literature review is too simple, and it can be extend on the importance and impact of multimodal transportation.

**We increased the number of references, made the classification of literature review more complete and clear, and expounded the innovative points of the paper more prominently in the paper. (2 Literature review)**

4. The panel data in Table 1 should be extended to the most recent.

**We updated the experimental data from 2017-2021 to 2017-2023, and re-regressed the model in the paper, and updated the data and results.**

5.  Regarding Table 2 and 3，the discussion is not enough. How to confirm that the impact of multimodal transport on the domestic circulation remains positive? Why is the impact significant, and the regression results are robust and reliable?”

**For Table 2 and Table 3, the analysis is supplemented. For the results presented by regression, *** represents the significance of the regression results, and the positive and negative coefficients represent the positive or negative effects of multimodal transport on the domestic and international double cycle and the degree of effect.**

6.  In Section 5.3, regarding the sentence "Drawing on the methodologies of relevant scholars", some appropriate literature should be cited to demonstrate that this is a viable methodology for this paper.

**In Section 5 and 3, we supplement the literature of the scholars used for reference in the section "learning from the methods of relevant scholars", in which the robustness testing methods such as tail shrinking and regression model replacement refer to the research of scholars such as Hu Hanhui and Gu Xiaoyan.**

7. In data preprocessing, there is a lack of description of how missing values are handled.

**In this paper, a small amount of missing data is supplemented by interpolation method or mean method. This description has been added to the text.**

8.  I suggest that limitations are discussed in Section 6.1 not 6.2.

**We have adjusted the restrictions and future outlook to section 6.1.**

Appended to this letter is our point-by-point response to the comments raised by the reviewers. The comments are reproduced and our responses are given directly afterward in a different bold type.

We would like also to thank you for allowing us to resubmit a revised copy of the manuscript. If there are any other [modifications](https://zhida.zhihu.com/search?content_id=238622203&content_type=Article&match_order=1&q=modifications&zhida_source=entity) we could make, we would like very much to modify them and we really appreciate your help.

On behalf of all the contributing authors, I would like to express our sincere appreciations of your letter and reviewers’ constructive comments concerning our article entitled “Research on the Impact Effect of Multimodal Transport on Domestic and International Dual Circulation: Evidence from China's Railway and Water Transport”. These comments are all valuable and helpful for improving our article.

Sincerely

2025.1.5
