# Supplementary material for: Research on the impact effect of multimodal transport on domestic and international dual circulation: Evidence from China’s railway and water transport
Source: PLoS One. 2025 Apr 25;20(4):e0319982. doi: 10.1371/journal.pone.0319982 (PMC12026926; doi:10.1371/journal.pone.0319982)
Supplement: Attachment — Submitted filename: Response_to_Reviewers_auresp_2.docx [file pone.0319982.s004.docx]

Dear Editor：

We are very grateful to your and the reviewers’ critical comments and thoughtful suggestions. Based on these comments and suggestions, we have made careful modification on the original manuscript. All changes made to the text are in red in the revised manuscript so that they may be easily identified. Some of your questions were answered below.

Once again, we acknowledge your comments and constructive suggestions very much, which are valuable in improving the quality of our manuscript.

Here are our responses to the reviewers’ comments one-by-one.

**Ⅰ. Reply to the comments of the first review expert**

1. In the literature review, network design should come first, followed by route optimization, and it is suggested that the order of section 2.1 and section 2.2 should be replaced.

**According to your suggestion, we have switched the order of Section 2.1 and Section 2.2. At present, it is 2.1 multimodal transport network design and 2.2 multimodal transport path optimization.**

2. The authors should recheck all the text expressions to avoid the clerical errors, for example, in Line 252, “Shanxi” is written as “Shaanxi”. In Line 275, there is no “、” in English.

**In line 252, examples are Shaanxi Province, China and the Xi 'an Assembly Center of China-Europe freight trains in Xi 'an, the capital of Shaanxi Province; Punctuation problem in line 275 has been corrected.**

3. In Line 299, “labor-intensive and export-oriented processing businesses” is mentioned. Is there any data to support whether labor-intensive industries should continue to dominate in the eastern part of China in the future? Because this seems to be inconsistent with the perception that labor-intensive industries are generally moving to the central and western regions, or even to other countries, please at least provide an explanation in the response.

**After reviewing relevant materials, we believe that there is indeed some deficiency in the statement "labor-intensive and export-oriented processing businesses" in line 299, which has been revised.**

4. In Line 469-473, the authors have made necessary additions to the provinces in the eastern, central and western regions, and it is suggested to summarize them in a table instead of too much text. Meanwhile, it is suggested to color the provinces in the east, central and west regions differently based on the blank map of China, which can significantly increase the readability and intuition.

**In Line 469-473, we have summarized the eastern, central and western provinces in the form of tables, and marked the eastern, central and western provinces with different colors on the basis of the blank map of China, which improves the readability and intuition of the article.**

5. In the structure of the article, it is suggested that the article's summary and limitations (section 6.1) should be placed after the policy suggestion (section 6.2).

**We have placed the summary and limitations of the article (section 6.1) after the policy recommendations (Section 6.2). (In Line 588-596)**

6. It is suggested that in response letter, add changes to the manuscript (with line numbers), with response to the review comments, in a different color.

**We adopted the revision mode for the revised part of the article, marked it with different colors, and added the specific line number of the modified content in the reply for your convenience.**

**Ⅱ. Reply to the comments of the second review expert**

1. In this paper, there are still some incorrect expressions about provinces. I hope they can be expressed in a standardized manner, such as "Shaanxi Province in China".

**We have fixed the syntax error you mentioned (In Line 252).**

2. Some basic formats of the article are not standardized. For example, line 173, no space between "al." and [23]; in table 9, prefer "domestic circulation" to "Domestic circulation", as all table headers are lowercase. I expect that authors check the full paper to improve the quality.

**In line 173, we have removed the excess Spaces; "Domestic circulation" in Table 9 (now Table 10) has been amended to "domestic circulation". We checked and corrected all these problems.**

Appended to this letter is our point-by-point response to the comments raised by the reviewers. The comments are reproduced and our responses are given directly afterward in a different bold type.

We would like also to thank you for allowing us to resubmit a revised copy of the manuscript. If there are any other [modifications](https://zhida.zhihu.com/search?content_id=238622203&content_type=Article&match_order=1&q=modifications&zhida_source=entity) we could make, we would like very much to modify them and we really appreciate your help.

On behalf of all the contributing authors, I would like to express our sincere appreciations of your letter and reviewers’ constructive comments concerning our article entitled “Research on the Impact Effect of Multimodal Transport on Domestic and International Dual Circulation: Evidence from China's Railway and Water Transport”. These comments are all valuable and helpful for improving our article.

Sincerely

2025.2.7
